# Conceptualizing the Humanized Hospital: A Multidimensional Textual Data Analysis from Undergraduate Nursing Students’ Perspectives

**DOI:** 10.3390/nursrep16020062

**Published:** 2026-02-13

**Authors:** Marika Lo Monaco, Gloria Littlemouse, Giuliano Anastasi, Ramona Gheorghe, Roberto Latina, Mariachiara Figura

**Affiliations:** 1 Department of Health Promotion Sciences, Maternal and Infant Care, Internal Medicine and Medical Specialties (PROMISE), University of Palermo, 90127 Palermo, Italy; marika.lomonaco@unipa.it (M.L.M.); roberto.latina@unipa.it (R.L.); mariachiara.figura@unipa.it (M.F.); 2 American Holistic Nurses Association (AHNA) Italian Chapter, 28040 Lesa, Italy; 3 Watson Caring Science Institute, Deerfield Beach, FL 33442, USA; 4 Centro di Eccellenza Mediterraneo per lo Sviluppo Accademico della Ricerca Infermieristica (CEMSIR), 80134 Naples, Italy; 5 Canadian Holistic Nurses Association, Ottawa, ON K2P 0E4, Canada; rgheorghe@quintehealth.ca; 6 Vanderbilt School of Nursing, Nashville, TN 37235, USA; gloria.littlemouse@vanderbilt.edu; 7 Department of Medicine and Surgery, University of Enna “Kore”, 94100 Enna, Italy; 8 Department of Clinical Operations, Quinte Health, Belleville, ON K8N 5A9, Canada

**Keywords:** humanized care, nursing education, undergraduate nursing students, textual data analysis, automatic analysis of textual data, exploratory multidimensional data analysis, student perspectives, hospital environment

## Abstract

**Background**: The humanization of care is increasingly recognized as a core component of healthcare quality; however, its meaning remains complex and strongly shaped by organizational, professional, and educational contexts. Nursing students, as future healthcare professionals, play a crucial role in the development and transmission of humanized care values, making their representations of the humanized hospital particularly relevant for understanding how these values are constructed during professional education. Aim: To explore how undergraduate nursing students conceptualize the humanized hospital. **Methods**: A qualitative exploratory study was conducted involving 742 undergraduate nursing students enrolled in a Bachelor of Science in Nursing program in Italy. Data were collected through a single open-ended written question inviting students to describe how they imagine a humanized hospital. Textual data were analyzed using Automatic Analysis of Textual Data within an Exploratory Multidimensional Data Analysis framework, enabling the identification of shared lexical patterns, discursive clusters, and latent semantic dimensions within a large textual corpus. **Findings**: Students articulated the humanized hospital as an integrated and system-oriented care environment in which relational, organizational, professional, and holistic dimensions are deeply interconnected. Humanization was associated not only with empathy, respect, and emotional engagement, but also with organizational functioning, teamwork, adequate resources, and professional competence. Two latent dimensions structured these representations: the first highlighted organizational systems as enabling conditions for person-centered care, while the second framed professional operability and technical competence as foundations for a holistic understanding of patients’ physical, psychological, and social well-being. **Conclusions**: Undergraduate nursing students’ discourse revealed an articulated and multidimensional representation of hospital humanization, conceptualizing it as an emergent property of healthcare environments rather than as a function of individual attitudes alone. These findings underscore the importance of addressing hospital humanization simultaneously at relational, educational, and organizational levels and highlight the need for nursing education programs and healthcare institutions to foster structural and professional conditions that sustainably support humanized care in clinical practice.

## 1. Introduction

The humanization of care is widely recognized as a core dimension of high-quality healthcare. It refers to a holistic, relational, and ethically grounded approach that acknowledges patients as persons rather than passive recipients of clinical interventions [1]. Within contemporary healthcare systems, the concept of humanization has progressively expanded from an individual professional attitude to a multidimensional construct encompassing relational, organizational, environmental, and ethical components.

Within nursing scholarship, humanized care is primarily framed through the lens of Caring Science. In particular, Jean Watson’s Theory of Human Caring conceptualizes humanization not as a structural attribute of healthcare organizations, but as a way of being in care, enacted through intentional, respectful, and authentic relationships between nurses and patients. Within this framework, caring is understood as a moral and relational process grounded in ethical intentionality, presence, and respect for human dignity, through which both patients and professionals are mutually influenced. Watson emphasizes that caring relationships create a healing environment that supports not only clinical outcomes but also personal meaning and well-being. From this perspective, humanization is not reduced to individual attitudes, but is embedded in professional practice and in the cultural and organizational contexts that enable or constrain caring encounters [2,3].

From this perspective, hospitals become humanized not solely through architectural design or procedural reforms, but through everyday caring encounters that are shaped by, and embedded within, broader organizational contexts [4].

International frameworks and empirical research further support this multidimensional understanding, emphasizing the role of empathy, person-centered communication, family involvement, and staff well-being as key elements of humanized healthcare environments [5,6]. At the same time, a substantial body of literature highlights the challenges of translating these principles into routine practice. High workloads, time pressure, inadequate infrastructure, and organizational cultures prioritizing efficiency over relational depth often constrain the enactment of humanized care, revealing tensions between ethical ideals and clinical realities [7,8].

In this context, nursing students represent a particularly relevant population for understanding how humanized care is conceptualized and internalized during professional education. As future healthcare professionals, students are simultaneously exposed to caring ideals promoted in academic curricula and to the organizational constraints encountered during clinical placements [9]. Research suggests that students’ perceptions of care evolve throughout training, influenced by clinical exposure, mentorship, and institutional culture [10,11]. However, less attention has been devoted to how students collectively conceptualize the idea of a “humanized hospital”.

Although humanized care is relevant across multiple healthcare settings, the hospital represents the primary context of professional socialization for undergraduate nursing students. Especially during the early years of training, the hospital is often their first and most intensive exposure to healthcare practice, shaping their initial representations of care, professionalism, and patient relationships. At the same time, the hospital constitutes a highly complex and symbolically charged organizational environment, where technological intensity, institutional routines, and patients’ vulnerability converge. For these reasons, it provides a particularly meaningful setting for exploring how nursing students imagine and construct humanization.

Most existing studies on humanized care in nursing education have relied on traditional qualitative approaches, such as interviews or focus groups, to explore students’ experiences, perceptions, and attitudes. While these methods provide rich and in-depth insights into individual meanings, they primarily capture subjective and situated accounts, often shaped by interaction with the researcher and by the narrative structure of the interview itself. As a result, less attention has been devoted to analyzing collective representations as discursive configurations that emerge across large student populations.

In this study, the focus is not on individual experiences of care, but on how the idea of a “humanized hospital” is collectively constructed and organized within students’ discourse. Automatic Analysis of Textual Data (AATD), embedded within an Exploratory Multidimensional Data Analysis framework, offers a distinctive methodological contribution by enabling the identification of latent semantic structures, shared lexical patterns, and multidimensional configurations of meaning within a large corpus of written narratives. Rather than privileging predefined categories or researcher-led coding, this approach allows meaning to emerge from the relational organization of language itself.

By adopting AATD, the present study addresses an underexplored gap in the literature, providing a population-level and system-oriented perspective on hospital humanization as a collectively constructed representation. This methodological choice enables moving beyond descriptive accounts of caring values and exploring how the relational, organizational, and professional dimensions of humanization are structurally interconnected within students’ shared discourse.

Accordingly, this study aims to explore how undergraduate nursing students conceptualize the humanized hospital by examining the latent semantic structures that organize their collective discourse, using Automatic Analysis of Textual Data within an Exploratory Multidimensional Data Analysis framework.

## 2. Materials and Methods

### 2.1. Study Design

This study employed a qualitative exploratory design to examine how undergraduate nursing students conceptualize a humanized hospital. Participants were students enrolled in a Bachelor of Science in Nursing program in Italy. Data were collected through a single open-ended written question designed to elicit students’ representations of what constitutes a humanized hospital environment.

Textual data were analyzed using Automatic Analysis of Textual Data (AATD) within the framework of Exploratory Multidimensional Data Analysis (EMDA), as proposed by Fraire (2009) [12]. This approach integrates quantitative statistical techniques with qualitative inquiry to identify latent semantic structures and discursive patterns within large textual corpora. An exploratory-descriptive perspective guided data interpretation, allowing systematic examination of collective representations embedded in students’ discourse [13].

In line with the study’s aim of providing a population-level overview of students’ representations of the humanized hospital, AATD was selected as a particularly informative analytical strategy for examining the overall semantic organization of the corpus and the latent dimensions shaping collective representations. The study is reported in accordance with the Consolidated Criteria for Reporting Qualitative Research (COREQ) checklist [14].

### 2.2. Setting and Recruitment

The study was conducted within a Bachelor of Science in Nursing program delivered across a main campus and affiliated teaching sites. All undergraduate nursing students enrolled in the program at the time of data collection were considered eligible for participation, including those from the first, second, and third academic years, as well as those not progressing within the standard time frame.

Recruitment followed a population-based approach aimed at capturing a comprehensive overview of students’ representations of the humanized hospital. Participation was voluntary and required written informed consent.

Given the population-based recruitment strategy and the study’s aim of mapping collective representations at a population level, the adequacy of the dataset was ensured through the inclusion of the entire eligible student population and through subsequent assessment of corpus stability and lexical adequacy in line with established criteria for AATD [15].

### 2.3. Data Collection

Data were collected through a single open-ended written question designed to explore students’ representations of the humanized hospital. Students were asked: How do you imagine a humanized hospital? No additional prompts or probes were used. The question was intentionally broad and non-directive, aiming to elicit spontaneous written narratives and individual representations related to the topic.

The question was administered via an online form (Google Forms), accessible through a QR code shared during in-person lectures. Prior to answering the open-ended question, participants were asked to provide basic sociodemographic and academic information, including gender (male/female/other), age (years), year of Bachelor’s degree enrollment (first/second/third/beyond the regular course duration), campus, and commuting status (commuter/non-commuter). All eligible undergraduate nursing students were invited to participate voluntarily, and written informed consent was obtained before data collection.

Responses were collected in the students’ original language (Italian) and were subsequently translated into English for data analysis.

Data collection took place in classroom settings, with facilitators present to provide technical support and to ensure appropriate conditions of privacy and autonomy during response writing. The average time required to complete the question was approximately 15–20 min.

### 2.4. Data Analysis

All responses were originally produced in Italian and subsequently translated into English to allow for uniform processing within the textual analysis software. The translation was performed by members of the research team with expertise in nursing research and proficiency in both Italian and English. To preserve semantic and contextual equivalence, the translation process followed established methodological recommendations for qualitative research [16]. Particular attention was paid to maintaining the original meaning of key concepts, relational expressions, and care-related terminology. When lexical or semantic ambiguities arose, translations were discussed collaboratively within the research team to reach a shared and conceptually coherent solution. This iterative process aimed to minimize semantic distortion while ensuring consistency across the corpus, thereby preserving the integrity of the lexical structures subjected to automated analysis.

Before applying multivariate analyses, a preliminary assessment of corpus adequacy and stability was conducted to ensure the reliability of the subsequent exploratory analyses. In accordance with established methodological recommendations for Automatic Analysis of Textual Data, this assessment relied on global lexical indicators commonly used to evaluate the robustness and internal coherence of large textual corpora (result section). These indicators were used as quality checks to verify that the corpus was sufficiently large, cohesive, and statistically stable to support the identification of shared lexical patterns and latent semantic structures. Rather than constituting analytical results per se, they ensured that the observed configurations reflected collective discursive regularities rather than artifacts of sparse or fragmented textual data [15].

Subsequently, several complementary techniques within the EMDA framework were applied to examine the structure of students’ discourse. First, similarity analysis was used to explore patterns of lexical co-occurrence and to identify central and peripheral semantic nuclei within the corpus. This step provided an initial overview of the semantic organization of the discourse and informed subsequent analyses.

Hierarchical Descending Classification (HDC), an unsupervised clustering technique, was then applied to partition the corpus into internally coherent lexical clusters. Each cluster grouped text segments sharing similar vocabularies and semantic orientations, thereby representing distinct ways in which students conceptualized the humanized hospital.

Finally, Principal Component Analysis (PCA) was employed to synthesize the results of the similarity and clustering analyses within a reduced factorial space. By projecting lexical forms and cluster structures onto a limited number of principal components, PCA facilitated an integrated interpretation of the latent semantic dimensions shaping students’ representations and the relationships between words, themes, and discursive profiles [17].

The combined use of Descending Hierarchical Classification and Principal Component Analysis represents a core feature of the Exploratory Multidimensional Data Analysis framework adopted in this study. While HDC enables the identification of internally coherent lexical classes based on shared vocabularies, PCA reduces the dimensionality of the lexical space by projecting these classes into a factorial space, enabling examination of their relational organization and the latent dimensions interpretable as synthetic axes summarizing the corpus.

The integration of these analytical procedures supports a multidimensional and relational interpretation of students’ discourse, moving beyond the identification of thematic clusters toward the exploration of underlying semantic oppositions and organizing principles. This combined approach enhances the interpretative depth of the analysis and would not be achievable through the application of a single analytical technique alone [12,15].

All analyses were performed using the open-source software IRaMuTeQ (version 0.7 alpha 2) [18].

### 2.5. Rigor

To ensure the quality and trustworthiness of this qualitative study, the criteria for rigor proposed by Lincoln and Guba (1985) [19] were applied and adapted to the AATD context.

Credibility was supported through a rigorous process of corpus construction, including the verbatim transcription of all open-ended responses, careful preparation of the textual material in accordance with IRaMuTeQ conventions, and joint verification by the research team to ensure internal consistency and semantic coherence. In addition, corpus adequacy and analytical reliability were supported through the assessment of empirically grounded lexical indicators, such as lexical balance parameters, which are widely recognized as essential prerequisites for multivariate textual analysis [13,15,20].

Transferability was enhanced by the inclusion of the entire undergraduate nursing student population, encompassing a wide range of academic experiences and perspectives. This broad and heterogeneous corpus enabled diverse representations of the humanized hospital, thereby increasing the potential relevance of the findings for other nursing education contexts with comparable organizational and educational structures.

Dependability was ensured through the transparency and traceability of the analytical process. All phases of the study, from corpus preparation to the selection and application of analytical techniques and the interpretation of results, were systematically documented, allowing the analytical pathway to be clearly reconstructed and, in principle, replicated [15].

Confirmability was supported using automated procedures for text segmentation and classification, which limited the direct influence of researchers’ preconceptions on the identification of lexical structures and patterns. At the same time, interpretation remained an essential component of the analytical process and was conducted through an iterative and dialogical approach involving constant comparison among researchers, in line with recommendations for the reflexive use of automated textual analysis [21].

Overall, the analytical approach adopted in this study remained firmly qualitative in its epistemological orientation. Quantitative indicators derived from multivariate statistical procedures were used exclusively as analytical tools to structure the textual corpus, identify salient lexical configurations, and support the interpretation of discursive patterns. Rather than producing numerical findings per se, these procedures functioned as heuristic devices that enhanced the transparency, stability, and interpretive grounding of the qualitative analysis. This approach enabled a systematic exploration of students’ representations while preserving the central role of qualitative interpretation in meaning construction [22,23].

### 2.6. Ethical Considerations

This study received approval from the Institutional Review Board of the University of Palermo (approval number: 342/2025). All procedures were conducted in accordance with internationally recognized ethical standards for research involving human participants, including the Declaration of Helsinki [24] and the ethical principles for health research. Participation was voluntary, and written informed consent was obtained from all participants before data collection. Data were collected anonymously, and no personally identifiable information was recorded.

## 3. Findings

### 3.1. Sample Characteristics

The sample comprised 742 undergraduate nursing students. Female students represented the majority, accounting for 69.5% of the sample.

The mean age of the included students was 23.59 years (SD = 5.06). The sample included undergraduate nursing students enrolled across different years of the Bachelor’s degree program. Most participants were in the first and second years of study, with 291 students (39.2%) enrolled in the first year and 294 students (39.6%) in the second year. Students in the third year accounted for 154 participants (20.8%). Only a very small proportion of the sample (n = 3; 0.4%) was enrolled beyond the regular duration of the program (Table 1).

### 3.2. Corpus Characteristics

The overall corpus, consisting of 742 responses to the open-ended question, comprised 25,818 occurrences (tokens). The type/token ratio was 6.91%, indicating a sufficiently robust structure to ensure stability in statistical processing, while maintaining good lexical diversity. The percentage of hapax (forms occurring only once) was 41.46%, well below the critical threshold of 50% suggested by Bolasco (2021) to avoid semantic dispersion [15]. Overall, these indices confirm the lexical quality of the corpus and its suitability for multivariate analysis (Table 2).

### 3.3. Multidimensional Analysis

#### 3.3.1. Similarity Analysis

**Figure 1:**
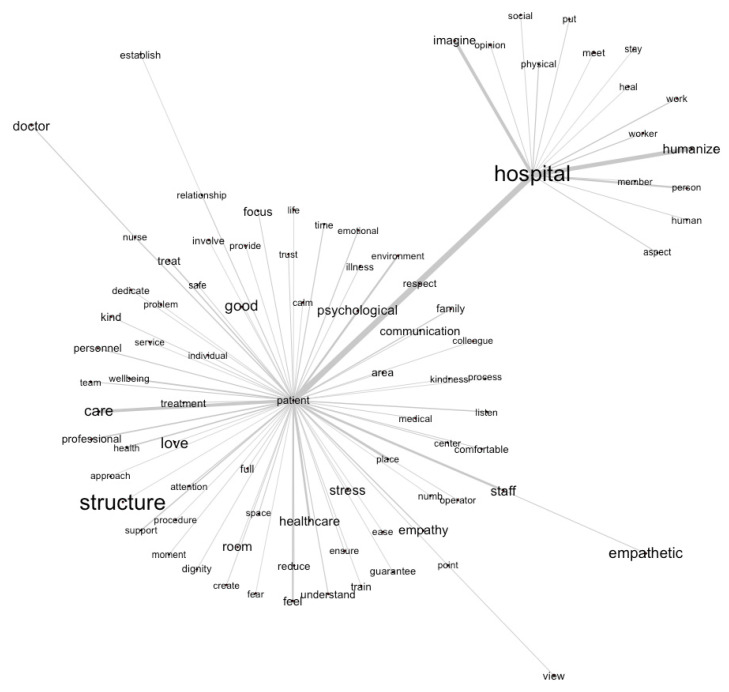
Similarity analysis network illustrating the main semantic structure underlying students’ representations of the humanized hospital. The network highlights the central role of the patient–hospital relationship and shows how relational, organizational, emotional, and ethical dimensions of care are interconnected within a shared discursive space.

Similarity analysis was conducted to explore patterns of lexical co-occurrence among the most frequent lemmas in the corpus and to identify the main semantic nuclei structuring students’ representations of the humanized hospital. The resulting network graph (Figure 1) represents lemmas as nodes and their co-occurrence relationships as edges, with thicker connections indicating stronger and more frequent associations.

At the core of the network, the strongest semantic association emerges between the lemmas patient and hospital, defining the primary axis around which the representation is organized. This central relationship indicates that students conceptualize the hospital primarily in relation to the patient, framing the healthcare environment as meaningful insofar as it is oriented toward the person receiving care. The hospital is thus represented not merely as a physical or organizational setting, but as a space whose identity is constructed through its relationship with patients.

From this central axis, multiple interconnected semantic fields unfold. The patient node functions as a major hub linking relational elements (e.g., empathy, communication, staff), technical and organizational aspects (e.g., treatment, healthcare, structure), emotional experiences (e.g., stress, feel), spatial features (e.g., room, place), and ethical values (e.g., respect, dignity, trust). The density and diversity of these connections highlight the integrated nature of students’ representations, in which interpersonal, organizational, emotional, and moral dimensions of care are closely intertwined.

Notably, the co-occurrence of care and treatment suggests that students distinguish between relational and technical dimensions of practice while simultaneously perceiving them as interdependent. Similarly, the association between patient and stress points to an awareness of the emotional burden inherent in hospitalization, reinforcing the perceived importance of supportive environments and empathic interactions. Within this network, staff and empathy emerge as closely connected to the patient node, underscoring the central role attributed to healthcare professionals and affective relationships in shaping the hospital experience.

The lemma respect occupies a pivotal position, acting as a semantic bridge between patient- and hospital-related vocabularies. Its central placement suggests that respect is perceived as a foundational value mediating the relationship between individuals and the healthcare institution. From an interpretative perspective, this configuration indicates that students do not conceptualize hospital humanization as a purely relational or emotional construct, but as an integrated system in which ethical values, professional roles, and organizational structures converge around the patient as the central reference point.

#### 3.3.2. Hierarchical Descending Classification

**Figure 2:**
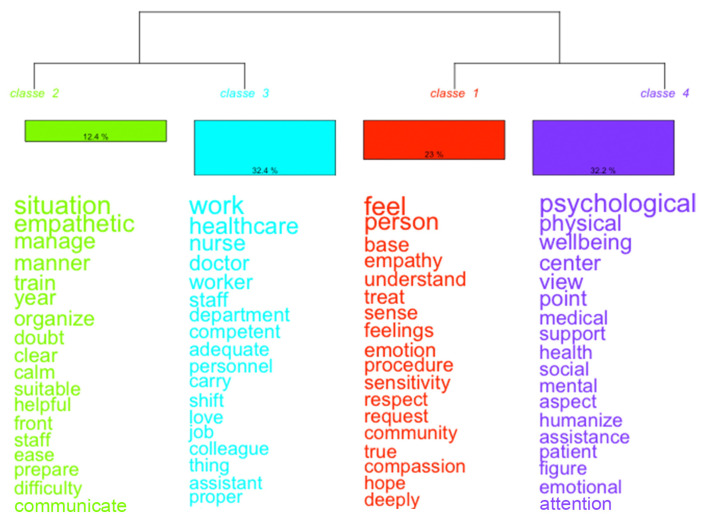
Descending Hierarchical Classification of the textual corpus, identifying four lexical classes that represent complementary perspectives through which students conceptualize hospital humanization, encompassing organizational, professional, relational, and holistic dimensions of care.

Hierarchical Descending Classification (HDC) was applied to identify internally coherent lexical classes within the corpus, resulting in four distinct classes that together accounted for 86.7% of the classified text segments. This high proportion indicates a well-structured and stable semantic organization of students’ discourse. The relative weight of each class was as follows: Class 2 (12.4%), Class 3 (32.4%), Class 1 (23.0%), and Class 4 (32.2%). The numerical ordering of classes reflects the statistical sequence generated by the algorithm and does not imply any conceptual or hierarchical prioritization (Figure 2).

Class 2 (Green Cluster; 12.4%), labeled “Empathic Management of Care Situations,” groups text segments emphasizing professionals’ capacity to manage complex clinical contexts with clarity, emotional regulation, and organizational competence. Humanization is framed here as the ability to combine empathic sensitivity with effective organization, preparedness, and training. This representation highlights care as a practice that requires both relational attunement and operational competence, suggesting that empathic engagement is perceived as inseparable from professional preparedness.

Class 3 (Blue Cluster; 32.4%), the largest cluster, reflects the organizational and professional context of hospital humanization. The lexical profile emphasizes professional roles, teamwork, departments, and system-level functioning, portraying the hospital as a complex organizational structure in which humanization emerges from coordinated action and role integration. In this perspective, care quality is not attributed to isolated individual behaviors but to the functioning of the healthcare system as a whole.

Class 1 (Red Cluster; 23.0%), labeled “Human and Emotional Relationship with the Patient,” captures the relational and affective dimension of care. The dominant vocabulary highlights empathy, respect, understanding, listening, and emotional sensitivity, framing humanization as the capacity to recognize patients as persons and to establish meaningful, emotionally attuned relationships. This class emphasizes interpersonal encounters as central moments in which humanization is enacted within the hospital setting.

Class 4 (Purple Cluster; 32.2%), labeled “Psychological Well-being and Holistic Perspective,” focuses on an integrated understanding of health that encompasses physical, psychological, and social dimensions. Humanization is represented as attention to overall well-being rather than to illness alone, reflecting a holistic orientation toward care that extends beyond clinical effectiveness to include emotional support and quality of life.

These four lexical classes delineate complementary perspectives through which students conceptualize the humanized hospital. Rather than representing isolated or competing dimensions, the clusters collectively articulate a systemic understanding of humanization as an emergent property of healthcare environments, shaped by the interaction between organizational structures, professional competence, relational engagement, and holistic attention to well-being.

#### 3.3.3. Principal Component Analysis

**Figure 3:**
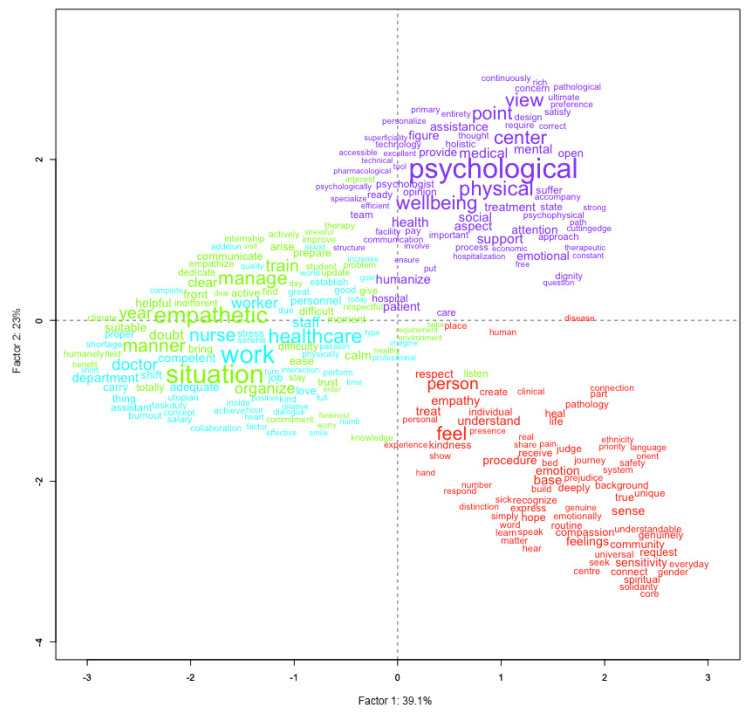
Principal Component Analysis factorial plane showing the relationships among the four lexical classes and the latent semantic dimensions structuring students’ representations of the humanized hospital. The figure illustrates how organizational systems and professional operability are interpreted as foundational conditions for person-centered and holistic care.

Principal Component Analysis (PCA) provides a reduced factorial representation of the latent semantic dimensions structuring students’ discourse on the humanized hospital. The projection of lexical forms and cluster profiles onto the factorial plane allows a global interpretation of relationships among the main semantic areas identified in the corpus (Figure 3).

The spatial distribution of the four clusters across the factorial plane confirms their semantic distinctiveness and complements the results of the hierarchical classification. The green and blue clusters occupy the left-central area of the space, highlighting the close association between organizational functioning and empathic management of care situations. The red and purple clusters are positioned on the right side of the plane, indicating a shared orientation toward the patient, while remaining differentiated along the vertical axis between emotional–relational and holistic well-being perspectives (Table 3).

The first two principal components accounted for 39.1% and 23.0% of the total inertia, respectively, explaining 62.1% of the overall lexical variance. This proportion supports a reliable interpretation of the major semantic oppositions underlying students’ representations.

The first axis (horizontal—39.1%), labeled “Organizational–System Orientation as a Condition for Person-Centered Care”, delineates a semantic continuum in which person-centered care emerges as dependent on the quality of the organizational and systemic context. On the left side of the axis, lexical forms related to organizational structure and daily management, such as structure, environment, work, situation, healthcare, team, and system, depict the hospital as an operational framework in which humanization is grounded in coordinated processes, adequate spaces, and professional organization. On the right side, the vocabulary shifts toward the patient and the emotional dimensions of care, with terms such as patient, care, feel, respect, illness, and process, reflecting an orientation toward relational proximity and recognition of the person’s lived experience. Rather than opposing these two poles, this axis suggests that, in students’ representations, person-centered care is conceived as achievable only when supported by an adequately humanized organizational system.

The second axis (vertical-23.0%), labeled “Professional operativity as a foundation for a holistic well-being perspective”, captures a semantic gradient reflecting how students integrate professional competence with broader conceptions of health and care. In the lower portion of the factorial space, lexical forms related to professional practice and situational operativity, such as training, skills, technical, teamwork, knowledge, and empathetic, are predominant. This pole represents a view of humanization grounded in the enactment of care through acquired competencies, clinical know-how, and the ability to manage care situations effectively within everyday practice. In the upper portion of the axis, the vocabulary shifts toward psychological and holistic dimensions of care, with terms such as psychological, physical, well-being, health, support, and social. This configuration depicts the humanized hospital as a space oriented toward integrated well-being, where attention extends beyond clinical effectiveness to encompass emotional, mental, and social dimensions of the person (Table 4).

The two principal components outline a coherent semantic framework in which representations of the humanized hospital are structured around the interplay between organizational systems and patient centrality, as well as between professional operability and holistic attention to well-being. Students’ representations indicate that professional operability is perceived as a necessary foundation upon which a holistic understanding of well-being can be constructed. In this sense, holistic care emerges not in opposition to technical expertise, but as its evolution and expansion within a humanized healthcare context. PCA thus provides an integrative reading of the corpus, confirming and refining the multidimensional structure emerging from the previous analyses.

## 4. Discussion

This study set out to explore how undergraduate nursing students conceptualize the idea of a humanized hospital, with the aim of shedding light on the meanings and assumptions that shape this concept during professional education. By focusing on shared discursive representations rather than individual experiences, the study provides insight into how hospital humanization is imagined as a multidimensional and system-oriented phenomenon. Overall, the findings reveal that students’ discourse articulates a coherent and integrated representation of humanization, in which the relational, organizational, professional, and holistic dimensions of care are deeply interconnected.

Several findings of the present study are consistent with international literature on humanized and person-centered care. In particular, students strongly emphasized relational and emotional components such as empathy, respect, attentive communication, and the recognition of patients as persons. These elements are widely identified as core features of humanized and person-centered care [3,5,6,25], and resonate closely with Watson’s Theory of Human Caring, which conceptualizes caring as an ethical and relational way of being grounded in intentionality, presence, and respect for human dignity [2,3]. The prominence of these dimensions indicates that ethical and relational values are central within students’ shared representations of care [26].

In line with previous studies, students also associated humanized care with attention to patients’ emotional experiences, vulnerability, and psychological well-being, reflecting a holistic understanding of health that extends beyond physical outcomes [27]. This orientation aligns with both Caring Science and person-centered care models, which emphasize the integration of emotional, psychological, and relational dimensions into clinical practice [2,6,9]. However, rather than framing holism as an abstract ideal, students situated it within the concrete conditions of hospital work, linking holistic care to professional competence and organizational functioning.

Beyond these convergences with prior research, the present study extends existing evidence through its strong systemic framing of hospital humanization. Consistent with contemporary person-centered care models, students conceptualized humanization as an emergent property of healthcare environments rather than as an individual moral attribute alone [5,6]. Humanized care was described as arising from the interaction between organizational structures, teamwork, leadership, and professional roles. This view resonates with evidence showing that nurses’ ability to deliver humanized care is profoundly shaped by organizational climate, workload, and institutional support [7,28,29].

The fact that such a systemic perspective is already articulated by undergraduate students suggests that organizational dimensions are salient [25,29,30] in their early professional socialization [31].

Professional competence emerged as another key dimension in students’ representations of a humanized hospital. Clinical knowledge, procedural competence, and professional confidence were seen as enabling nurses to be more emotionally present, attentive, and responsive to patients. These findings challenge traditional accounts of the theory–practice gap [32,33] by indicating that, within students’ collective representations, hard and soft skills are integrated into a unified conception of humanized care [34].

From a theoretical perspective, these findings both confirm and extend Caring Science. While Watson’s theory places primary emphasis on the nurse–patient relationship as the locus of humanization [3], students’ representations highlight that caring encounters are deeply embedded in organizational and educational contexts. Humanized care thus emerges not only from ethical intentions, but from the alignment of values, competencies, and institutional structures [35]. This integrative view is consistent with contemporary person-centered care frameworks [5,6] and supports a systemic understanding of humanization that bridges relational ethics with organizational and professional realities.

Overall, the study provides a theoretically grounded account of how future nurses imagine hospital humanization as a dynamic configuration of relational, professional, and organizational processes. By mapping students’ shared representations onto established frameworks of Caring Science, person-centered care, and holistic nursing, the findings contribute to a more integrated understanding of how humanized care is conceptualized and potentially enacted in contemporary hospital settings.

### 4.1. Strengths and Limitations

The study presents several strengths. Involving the entire undergraduate nursing student population, it provides a comprehensive and inclusive overview of how hospital humanization is collectively constructed during professional education. This population-level approach enables the identification of shared meanings and latent semantic structures that may remain less visible in smaller-scale qualitative studies. In addition, the use of AATD allowed for a systematic and transparent exploration of a large qualitative corpus, supporting the identification of robust discursive patterns while preserving interpretive depth and reducing the risk of selective focus on isolated narratives.

Some limitations should nevertheless be acknowledged. The study was conducted within a single national and educational context, which may limit the transferability of findings to healthcare systems characterized by different organizational cultures or educational models. Secondly, although responses were originally collected in Italian, textual analysis was conducted on an English-translated corpus to ensure the interpretability of graphical outputs and lexical representations for an international readership; while the translation process was carefully managed to preserve semantic equivalence, subtle language-specific nuances may have been attenuated, representing a methodological trade-off of the study. Moreover, while students’ written responses offered rich insights into collective representations, they did not allow for in-depth exploration of individual experiences or meanings that might have emerged through interviews or focus groups. It is important to note that these findings do not constitute an assessment of individual students’ cognitive sophistication or clinical maturity. Rather, they describe the semantic and relational organization of a large corpus of textual data, reflecting how meanings related to hospital humanization are collectively constructed within a specific educational and cultural context. The observed complexity, therefore, refers to the structure of the shared discourse, rather than to individual psychological attributes. Finally, the cross-sectional design captures students’ representations at a specific moment in their educational trajectory and does not permit examination of how these conceptualizations may evolve.

### 4.2. Implications for Nursing

The findings of this study have relevant implications for nursing education, clinical practice, and healthcare organizations. By showing that students’ representations of the humanized hospital are structured around the interaction between organizational systems, professional operability, and relational care, the results suggest that humanization should be addressed as a systemic and educational priority, rather than as an individual disposition alone.

For nursing education, the identified semantic dimensions provide a useful heuristic framework for curriculum design and reflective learning. Educational programs that integrate organizational analysis, ethical reflection, and clinical training may support the development of a professional identity that is both technically competent and humanistically grounded. Teaching strategies such as simulation, reflective writing, and guided clinical debriefing could be explicitly oriented toward exploring these interconnections.

For clinical practice and organizational leadership, the findings highlight that humanized care depends on supportive work environments, adequate staffing, teamwork, and clear organizational structures. Healthcare institutions should therefore view humanization not only as a matter of interpersonal behavior, but also as an outcome of organizational design and leadership. Initiatives aimed at improving humanized care may benefit from addressing workflow organization, interprofessional collaboration, and professional development alongside relational skills.

Healthcare organizations may benefit from involving students and early-career professionals in discussions about humanization, as their perspectives reveal both ethical aspirations and pragmatic awareness of systemic constraints. Such engagement could inform the development of policies and interventions aimed at aligning organizational processes with humanized care values.

At a conceptual level, the two latent semantic dimensions identified through PCA can serve as a heuristic model for understanding how humanization is enacted in hospital settings. This model links organizational–system conditions with person-centered orientations, and professional operability with holistic well-being, offering a framework that may guide future research, educational interventions, and organizational evaluation tools.

Future studies could extend this work by comparing students’ representations across different educational systems or cultural contexts, or by adopting longitudinal designs to explore how these representations evolve throughout training and into professional practice. Combining automated textual analysis with in-depth qualitative methods may also provide complementary insights, allowing researchers to connect shared semantic structures with individual lived experiences.

More broadly, the study demonstrates the value of exploring humanization through the perspectives of future professionals. Understanding how students conceptualize the humanized hospital offers a unique window into the values and assumptions that will shape the future of nursing practice and healthcare organizations.

## 5. Conclusions

This study explored how undergraduate nursing students conceptualize the humanized hospital, highlighting a multidimensional representation of humanization as a systemic, professional, and relational phenomenon. Students’ shared discourse portrays the humanized hospital not simply as an interpersonal ideal, but as an integrated care environment in which organizational structures, professional competence, and ethical–relational values are closely interconnected.

The findings show that person-centered care is represented as dependent on supportive organizational conditions, while professional operability is viewed as a foundation for holistic attention to patients’ physical, psychological, and social well-being. Humanization thus emerges as a dynamic configuration linking structure and relationship, technical expertise and ethical commitment, and individual care encounters with institutional responsibility.

By foregrounding students’ collective representations, this study highlights the importance of addressing hospital humanization simultaneously at relational, educational, and organizational levels. These findings support the need for nursing education programs and healthcare institutions to foster not only caring values, but also the structural and professional conditions that enable humanized care to be enacted in everyday clinical contexts.

## Figures and Tables

**Table 1:** Participants’ characteristics.

Variable (Range)	N	%	Mean	SD (±)	Min	Max	Median
Gender							
Female	516	69.5					
Male	224	30.2					
Other	2	0.3					
Age, years			23.59	5.06	19.00	48.00	22.00
Year of Bachelor’s degree enrollment							
First	291	39.2					
Second	294	39.6					
Third	154	20.8					
Beyond the regular course duration	3	0.4					
Course section/Campus							
Campus Palermo, Course Section 1 (Gordon)	270	36.4					
Campus Palermo, Course Section 1 (Nightingale)	143	19.3					
Campus of Agrigento	54	7.3					
Campus of Caltanissetta	105	14.2					
Campus of Trapani	170	22.9					
Commuting status							
Commuter students	343	46.2					
Non-commuter students	399	53.8					
Total	742						

**Table 2:** Descriptive and lexical indicators are used to assess corpus adequacy and stability before multivariate textual analysis, supporting the reliability of the subsequent exploratory analyses.

Item	Value
Number of texts	742
Number of occurrences	25,818
Number of forms	1785
Number of hapax	740 (2.87% of occurrences–41.46% of forms)
Mean number of occurrences per text	34.80

**Table 3:** Overview of the four lexical classes identified through Descending Hierarchical Classification and their positioning within the PCA factorial space, illustrating the main semantic orientations structuring students’ representations of the humanized hospital.

HDC Class	Semantic Focus	Dominant Dimensions	Position in Factorial Space
Class 2—Green (12.4%)	Empathic management of care situations	Organizational–operational/Professional competences	Left-central, lower
Class 3—Blue (32.4%)	Organizational and professional context	System-oriented functioning	Left-central
Class 1—Red (23%)	Emotional and relational care	Patient-centered emotional orientation	Right lower
Class 4—Purple (32.2%)	Psychological well-being and holistic care	Integrated well-being perspective	Right upper

**Table 4:** Latent semantic dimensions emerging from Principal Component Analysis, highlighting the interaction between organizational, professional, relational, and holistic perspectives in students’ conceptualization of the humanized hospital.

Dimension (%)	Semantic Pole A	Semantic Pole B	Interpretative Interaction
Organizational–System Orientation as a Condition for Person-Centered Care (39.1%)	Organizational—system orientation	Person-centered relational orientation	Humanization emerges from the alignment between organizational structures and relational care, rather than from either dimension alone
Professional operativity as a foundation for a holistic well-being perspective (23%)	Professional operativity	Holistic well-being perspective	Professional competence is perceived as meaningful when integrated into a broader, holistic understanding of patient well-being

## Data Availability

The data presented in this study are not publicly available due to ethical and privacy restrictions, but may be available from the corresponding author upon reasonable request.

## Public Involvement Statement

No public involvement in any aspect of this research.

## Guidelines and Standards Statement

This manuscript was drafted in accordance with the Consolidated Criteria for Reporting Qualitative Research (COREQ) checklist for qualitative research [14].

## Abbreviations

The following abbreviations are used in this manuscript:

AATD	Automatic Analysis of Textual Data
EMDA	Exploratory Multidimensional Data Analysis
TTR	Type-Token Ratio
GDPR	General Data Protection Regulation
EU	European Union
COREQ	Consolidated Criteria for Reporting Qualitative Research
HDC	Hierarchical Descending Classification
PCA	Principal Component Analysis
ECU	Elementary Context Unit
